# The Aarhus statement: improving design and reporting of studies on early cancer diagnosis

**DOI:** 10.1038/bjc.2012.68

**Published:** 2012-03-13

**Authors:** D Weller, P Vedsted, G Rubin, F M Walter, J Emery, S Scott, C Campbell, R S Andersen, W Hamilton, F Olesen, P Rose, S Nafees, E van Rijswijk, S Hiom, C Muth, M Beyer, R D Neal

**Affiliations:** 1 Centre for Population Health Sciences, University of Edinburgh, Edinburgh, UK; 2 Research Unit for General Practice, Aarhus University, Aarhus, UK; 3 School of Medicine and Health, Durham University, Stockton-on-Tees, UK; 4 General Practice and Primary Care Research Unit, University of Cambridge, Cambridge, UK; 5 School of Primary, Aboriginal and Rural Health Care, University of Western Australia, Perth, Western Australia, Australia; 6 Unit of Social and Behavioural Sciences, Dental Institute, King's College London, London, UK; 7 Primary Care Diagnostics, Peninsula College of Medicine and Dentistry, Exeter, UK; 8 Department of Primary Health Care, University of Oxford, Oxford, UK; 9 North Wales Centre for Primary Care Research, Bangor University, Bangor, UK; 10 University Medical College, St Radboud, Nijmegen, The Netherlands; 11 Cancer Research UK, London, UK; 12 Institute for General Practice, Johann Wolfgang Goethe University, Frankfurt, Germany

**Keywords:** early diagnosis, methods, definitions, diagnostic intervals

## Abstract

Early diagnosis is a key factor in improving the outcomes of cancer patients. A greater understanding of the pre-diagnostic patient pathways is vital yet, at present, research in this field lacks consistent definitions and methods. As a consequence much early diagnosis research is difficult to interpret. A consensus group was formed with the aim of producing guidance and a checklist for early cancer-diagnosis researchers. A consensus conference approach combined with nominal group techniques was used. The work was supported by a systematic review of early diagnosis literature, focussing on existing instruments used to measure time points and intervals in early cancer-diagnosis research. A series of recommendations for definitions and methodological approaches is presented. This is complemented by a checklist that early diagnosis researchers can use when designing and conducting studies in this field. The Aarhus checklist is a resource for early cancer-diagnosis research that should promote greater precision and transparency in both definitions and methods. Further work will examine whether the checklist can be readily adopted by researchers, and feedback on the guidance will be used in future updates.

## Background

Early diagnosis of symptomatic cancer is considered to be central to the achievement of better outcomes (Richards, 2009). There are national initiatives in many countries with the intention of achieving early presentation with symptoms and early diagnosis through more effective diagnostic routes. For example, the English National Awareness and Early Diagnosis Initiative (NAEDI) (Richards, 2009) is a programme of work intended to achieve early presentation of people with symptoms to optimise clinical practice and to improve GP access to diagnostics. Similar important initiatives are being undertaken in a growing number of jurisdictions (Rubin *et al*, 2011).

Variations in cancer survival between countries of comparable affluence and health systems have prompted international efforts to better understand these differences, often with a focus on the role of early diagnosis (Coleman *et al*, 2011). The scope for interventions to improve early diagnosis is wide and is receiving significant investment by the national governments. Several initiatives have acknowledged the need for complementary programmes of research in order to gain better understanding of patient and clinician behaviour and to determine the effectiveness of the interventions they are funding (Cancer Reform Strategy, 2007; Olesen *et al*, 2009).

Research in this area is, however, complex; the pathways to cancer diagnosis are often not straightforward and rarely linear (Fisher *et al*, 2010; Jordan *et al*, 2010). The health-system context in which research is carried out has a major influence on individual stages of these pathways. Moreover, the published research to date is characterised by a wide range of methodological approaches, often poorly or imprecisely described and with little theoretical basis. A wide range of methods have been used to measure time points and intervals, often dictated by the available data and resources (Neal, 2009). As a result, research findings are frequently difficult to interpret and typically not comparable beyond individual countries.

Furthermore, a number of different theoretical and methodological perspectives have been applied to the definition and measurement of time points and intervals in early diagnosis research, and these perspectives are often not explicitly delineated in the literature to date. Biomedical approaches predominate – they imply a direct relationship between disease and symptoms in the presence of illness. Other perspectives are less well-integrated within the literature; psychological approaches distinguish between ‘bodily changes’ (both sensations and visible changes) and ‘symptoms’ and are interested in the processes by which one becomes the other (Teel *et al*, 1997; Van Wijk and Kolk, 1997; Bradley *et al*, 2001). Sociological and anthropological approaches explore the processes by which bodily sensations are interpreted as symptoms; they are predicated on the view that symptoms evolve and are interpreted through the relationship between the individual and a given social and cultural context (Alonzo, 1984).

**Figure 1:**
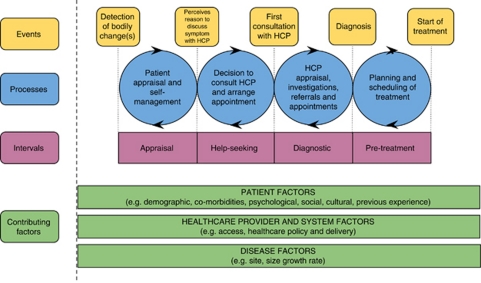
Model of pathways to treatment (Walter *et al*, 2011).

In the light of different purposes of the research, several theoretical models have been developed to describe the events and processes that underpin the pathway to symptomatic cancer diagnosis. Influential among these are the Danish model developed by Olesen *et al* (2009) and the model of pathways to treatment (see Figure 1), a refinement by Walter *et al* (2011) of the Andersen Model of Total Patient Delay.

Hence, early diagnosis research is characterised by its complexity, a poorly developed set of definitions and methodological tools, a lack of transparency in disciplinary perspectives and an absence of a widely-accepted underlying theoretical model. Accordingly, in order to advance the quality and consistency of studies of diagnostic intervals in symptomatic cancer, an international Consensus Working Group (CWG) was convened in November 2009. The purpose of the group was to formulate definitions of key time points and to make methodological recommendations for researchers in this field. The work was commissioned by the Cancer Research UK and the Department of Health in England and undertaken under the auspices of the Cancer and Primary Care Research International (Ca-PRI) Network (http://www.ca-pri.com/).

The aim of this paper is to propose and discuss a standardised set of definitions that can be used in research on early cancer diagnosis, relating to key time points and intervals in early diagnosis research. We also present methodological recommendations, principally on data collection and analysis.

## Methods

We used a ‘consensus conference’ approach (Durocher, 1994) that encompasses consensus meetings, presentations and wide circulation of draft outputs for modifications and refinement. It has been used in other fields to produce guidance for researchers and clinicians, and is particularly useful when a wide range of clinical, sociological, cultural and other perspectives need to be incorporated within the guidance. We also drew on nominal group techniques (Black *et al*, 1999) in our consensus meetings and in the exchange of information in the intervals between the meetings.

Members of the CWG were drawn from a range of disciplinary and methodological backgrounds including primary care, health-service research, social science, psychology, epidemiology and statistics. The CWG was supported by an international expert reference group comprising individuals from a wide range of clinical and methodological backgrounds. The CWG met three times between January and November 2010 in London and Aarhus. Each of the meetings comprised a series of presentations addressing theoretical, empirical and methodological areas of the project; the ensuing discussions were recorded and circulated between meetings. Consensus was achieved through an iterative approach, whereby proposed statements and checklists were developed and refined in the course of these meetings and informed by the presentations. Each element of the statement was discussed in detail by all members of the group until consensus was reached. The draft statement was then reviewed by an expert reference group and further refined in the light of their feedback.

The CWG had a close familiarity with the literature in this field. Nevertheless, we decided to underpin this by using systematic approaches to identify and assess relevant literature relating to existing questionnaires and survey tools. Accordingly, we further examined 279 included papers from a concurrent systematic review, examining the association between outcomes and diagnostic intervals (Neal, 2011). The inclusion criteria are shown in Box 1.

## Results

After examining the early cancer-diagnosis literature and taking into account the extensive theoretical work in this field, the CWG concluded that:

- There is little consistency in the definition and measurement of key time points and intervals;
- There is little guidance for researchers in designing studies that require the measurement of diagnostic time points and intervals;
- Little work in this field explicitly uses a theoretical framework to underpin definitions and measurement of diagnostic intervals;
- There is a lack of transparency and precision over methods and instruments in early diagnosis research – typically, important study elements, such as instrument development, are poorly described.

Accordingly, the CWG group developed the ‘Aarhus Statement’ comprising the following series of definitions and recommendations:

### Definitions

**Figure 2:**
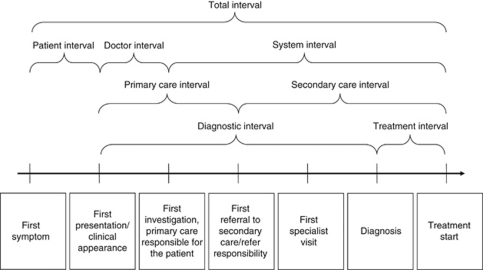
An illustration of the overall milestones and time intervals in the route from first symptom until start of treatment (Olesen *et al*, 2009).

The relevance of individual time points and intervals varies between health-care systems. Nevertheless, in an international context, the importance of the following four time points emerged in the Consensus Working Group discussions – we found the representation in Figure 2, illustrating the time points and their associated intervals, helpful:

#### Date of first symptom

This should be defined as ‘the ‘time point when first bodily changes and/or symptoms are noticed’. Researchers should consider this definition to encompass several key components: the date when the first bodily change was noticed, the date when the first symptom was noticed, the date when the person perceives a reason to discuss the symptom with a health-care professional and the date when the first ‘alarm’ or ‘high-risk’ symptom was noticed. Researchers should report clearly as to which of these definitions of first symptom has been applied in their study.

The term ‘patient delay’ should no longer be used – instead, ‘appraisal interval’ (time taken to interpret bodily changes/symptoms) and ‘help-seeking interval’ (time taken to act upon those interpretations and seek help) are more helpful in describing the ‘patient interval’ (see Figure 1). Researchers should acknowledge in their definition as to how they have dealt with the complexity of this time point, for example, where symptoms are common, nonspecific, multiple or chronic, where other morbidities coexist, and the social and health care context. It is important to recognise that often symptoms are medically defined, and such definitions may be inconsistent with lay-symptom definitions.

#### Date of first presentation

This should be considered ‘the time point at which, given the presenting signs, symptoms, history and other risk factors, it would be at least possible for the clinician seeing the patient to have started investigation or referral for possible important pathology, including cancer’. There should be a demonstrated understanding of the pattern of symptoms in the lead up to the first presentation (e.g., frequency, chronicity and presence of other symptoms) and whether the date has been defined from the perspective of the health-care provider or the patient. There should be precise descriptions of where this first presentation occurs (e.g., primary care, hospital department and so on).

#### Date of referral

This should be considered ‘the time point at which there is a transfer of responsibility from one health-care provider to another (typically, in ‘gatekeeper’ health-care systems, from a primary care provider to a doctor/service specialising in cancer diagnosis and management) for further clinical diagnostic and management activity, relating to the patient's suspected cancer’. Patients may be referred more than once or between specialists; there are risks of cross-referrals within secondary care and complex diagnostic routes (where patients ultimately receive treatment from specialist services that are different to those initially targeted). Researchers should use a consistent and explicit method for dealing with such complexities. Further, referral for investigations should be considered as a subsidiary time point that may be of significance in some health-care settings but is not equivalent in cases where actual responsibility for patient management is not transferred. The service targeted in the referral should also be described.

#### Date of diagnosis

Studies reporting any time interval that either begins or ends with ‘diagnosis’ should be explicit about how that date is measured and what it actually means with respect to the diagnostic journey within that health system. Researchers should consult the well-developed hierarchical rationales available in the public domain in choosing their definition of date of diagnosis – one example is the hierarchy produced by the European Network of Cancer Registries (http://www.encr.com.fr/incideng.pdf) which is shown in Box 2.

### Methodological approaches

Most studies on early cancer diagnosis involve retrospective data collection, which can be subjected to recall bias (Friedman *et al*, 2005). Prospective studies on patients with symptoms are often recommended (Summerton, 2002), although they pose difficulties due to the large numbers of participants required. Time since diagnosis is a critical sampling issue – sampling too long after diagnosis increases the risk of recall bias and increases the likelihood of attrition due to death or terminal illness. Conversely, sampling too soon after diagnosis may be insensitive and may increase the likelihood of attrition due to ongoing active treatment (surgery, chemotherapy and radiotherapy). The sampling method, whether through a cancer registry, hospital-based sampling or other mechanism, should be reproducible and thoroughly described.

#### Primary data collection from patients and/or providers

The key challenge in collecting data on diagnostic intervals is to obtain valid and complete data and, at the same time, capture the complexity of the pathways to a diagnosis of cancer. However, this might lead to overly cumbersome approaches and significant response burden. Therefore, researchers should develop questions which are (1) precise about the time point they are endeavouring to describe; (2) applicable to both the cancer in question and the likely symptom and symptom complexes that the patient experiences; and (3) specific about the context of the health-care system.

Questions should be derived from a clearly stated theoretical basis, and the health-care context should be clearly described, to allow for appropriate interpretation of responses. Ways in which the measurement approach takes into account multiple symptoms, chronic symptoms and co-existent co-morbidity should be described.

For measurement of the date of first symptom and date of first presentation, in-depth qualitative interviews with patients are preferred when there is a need for detailed understanding of the time point. Strategies such as calendar landmarking (Glasner and Van der Vaart, 2009) should be considered to reduce recall bias in qualitative interviews. In common with research in other fields, patient-completed surveys (typically in the form of self-completion questionnaires) provide the best opportunity for producing large, population-based data sets, but their limitations in capturing the complexity of these time points should be acknowledged and researchers should clarify how the survey has been developed in accordance with the international standards (Mokkink *et al*, 2010).

For the date of first presentation, ideally, information should be gathered from both patients and primary-care providers, as their concepts of this time point may differ, particularly in the context of vague, nonspecific or chronic symptoms. Open-ended questions, while making coding more difficult, are typically needed to encompass the complexity of cancer-related symptoms.

It is important to scrutinise the literature to be sure that projects include the best available measurement instrument. In future, we might see internationally validated standard questionnaires in this field. Therefore, it is also important that researchers provide very transparent information on development of interviews and surveys.

#### Case-note audit

Retrospective examination of case notes (e.g., charts and medical records) is an important source of information in medical research and clinical audit; a vital consideration is the accuracy of this information (Lilford *et al*, 2007). This approach should ideally be used to augment or validate data from other sources, particularly for complex time points such as date of first symptom. There should be a description of the process of how the clinician makes and codes the record and how clinicians interpret and record the patient's history in the clinical notes. Further, the likely completeness of the case-note data should be described. There is typically little data in secondary case notes on early stages of the diagnostic pathways (i.e., time points before the patient arrives in secondary care), although they are an important source of information on latter stages of the patient's journey.

#### Primary-care database analysis

There has been a growth in the number and quality of databases, which collect encounter and diagnostic information from primary care – one example is the General Practice Research Database (GPRD) (Jones *et al*, 2009). These offer the potential to conduct analyses on large samples and can facilitate study designs such as retrospective cohort studies. When used, there should be a thorough description of the database and its capacity to capture valid information on time points and intervals (e.g., completeness and accuracy of recording of encounter data). Further, the limitations deriving from coding systems used in the database should be described and there should be the standard procedures for both conceptualising data fields and uploading data in a standard format. In common with case-note audit, there are limitations in deriving information on time points, such as date of first symptom from analysis of databases, although both audit and database analysis can be useful in deriving information on the date of first presentation.

### The Aarhus checklist

Based on these recommendations, the CWG has developed a checklist for early diagnosis researchers. The checklist is intended for individuals undertaking research that involves the description and measurement of intervals in the cancer diagnostic journey. It has been produced with the intention of promoting greater consistency and transparency in methods and measurements. Furthermore, it is a resource both for those developing studies that require measurement of intervals and/or mapping of cancer patient journeys and for journal paper reviewers, editors and funding bodies who can use these items as a framework for assessing the quality of research applications and papers.


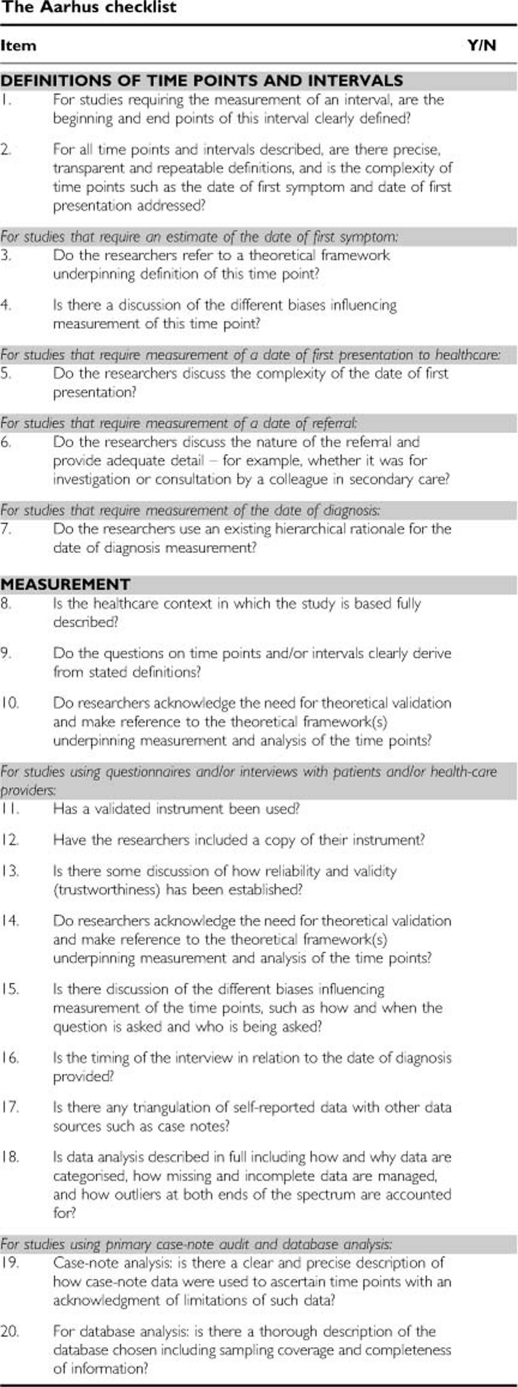


## Discussion

We report the first comprehensive guidance for the conduct and reporting of research in this field. We have highlighted the many challenges and pitfalls in early diagnosis research, and call for greater precision and transparency in both definitions and methods. Our rigorous and multidisciplinary approach to consensus development, coupled with detailed analysis of the existing early diagnosis literature, has produced guidance that can promote (the primary) consistency and methodological rigour in this field. Our recommendations are relevant to researchers, funding bodies, and journal editors and reviewers. The next step is to examine whether these definitions and recommendations can be readily adopted by researchers; evidence from similar previous initiatives suggests that researchers are receptive to new methodological guidance and that it can have a positive effect on research quality (Willis and Quigley, 2011; Wynne *et al*, 2011).

Although our checklist has not addressed analytical techniques, the choice of technique is crucial, given that analyses of time intervals are used to monitor quality of clinical trajectories and in research on prognosis of cancer. In prognostic research, there is strong evidence to suggest that time-interval data should be analysed in a statistical model using time interval as a continuous variable, rather than a dichotomised model, in order to minimise bias arising from what has been termed ‘the waiting time paradox’ (Crawford *et al*, 2002; Torring *et al*, 2011).

We have identified a pressing need for more methodological work in this field and we are in the process of further evaluation and validation of our recommendations, which can be viewed in greater detail on the Cancer and Primary Care Research International (Ca-PRI) Network (http://www.ca-pri.com/). Feedback on the guidance will be used for future updates.

## Conclusion

There are growing international efforts to describe and measure patient journeys prior to a cancer diagnosis. Accurate descriptions of these patient journeys and valid measurements of diagnostic intervals are essential to determine the effectiveness of interventions to reduce them. The Aarhus checklist will facilitate the standardised and uniform definition and reporting of studies in this area.

## Inclusion Criteria for Systematic Review

*A study or analysis was included in the review if it fulfilled all the criteria below:*

 Participants: only studies or analysis that examined symptomatic cancer patients presenting with primary cancers were included.

 Aim of study: ideally, to analyse the impact of the interval between the time of first symptomatic presentation and definitive diagnosis or treatment on a health-related outcome.

 Outcome: any study, analysing any health-related outcome, was considered for inclusion; these include survival, diagnostic stage, treatment assignment after diagnosis and other measures of ‘curability’. Survival was the main outcome of interest; additionally, studies examining health-related quality-of-life, resource-use and psychological outcomes were considered for inclusion.

 Study design: there were no restrictions on the types of study design that were considered for inclusion.

## European Network of Cancer Registries: Hierarchy for Defining the Date of Diagnosis (http://www.encr.com.fr/incideng.pdf)

*In the order of declining priority:*

1. Date of first histological or cytological confirmation of this malignancy (with the exception of histology or cytology at autopsy). This date should be, in the following order:

 (a) date when the specimen was taken (biopsy)

 (b) date of receipt by the pathologist

 (c) date of the pathology report

2. Date of admission to the hospital because of this malignancy.

3. When evaluated at an outpatient clinic only: date of first consultation at the outpatient clinic because of this malignancy.

4. Date of diagnosis, other than 1, 2 or 3.

5. Date of death, if no information is available other than the fact that the patient has died because of a malignancy.

6. Date of death, if the malignancy is discovered at autopsy.
